# Level and comfort of caregiver–young adolescent communication on sexual and reproductive health: a cross-sectional survey in south-western Uganda

**DOI:** 10.1186/s12889-022-14561-3

**Published:** 2022-11-19

**Authors:** Cecilia Akatukwasa, Viola N. Nyakato, Dorcus Achen, Elizabeth Kemigisha, Daniel Atwine, Wendo Mlahagwa, Stella Neema, Gad Ndaruhutse Ruzaaza, Gily Coene, Godfrey Z. Rukundo, Kristien Michielsen

**Affiliations:** 1 International Centre for Reproductive Health, Department of Public Health and Primary Care, Faculty of Medicine and Health Sciences, Ghent University, Ghent, Belgium; 2 Faculty of Interdisciplinary Studies, Mbarara University of Science and Technology, P.O. Box 1410, Mbarara, Uganda; 3 Nordic Africa Institute, Uppsala, Sweden; 4 Centre of Expertise Gender, Diversity and Intersectionality, Vrije Universitet Brussels, Brussels, Belgium; 5 Department of Community Health, Mbarara University of Science and Technology, P.O. Box 1410, Mbarara, Uganda; 6 Department of Sociology and Anthropology, Makerere University, P. O. Box 7062, Kampala, Uganda; 7 Department of Psychiatry, Mbarara University of Science and Technology, P.O. Box 1410, Mbarara, Uganda

**Keywords:** Sexual and reproductive health, Caregiver–child communication, Caregiver, Young adolescent, Sexuality education, Uganda

## Abstract

**Background:**

Communication on sexual and reproductive health (SRH) between caregivers and their young adolescent children plays a significant role in shaping attitudes and behaviours that are critical to laying the foundations for positive and safe SRH behaviours in later adolescence. Nevertheless, this communication is often limited, particularly in countries where adolescent sexuality is taboo. This study assessed the topics discussed (‘level’) and the comfort of caregivers with communicating with young adolescents on SRH, and their correlates.

**Methods:**

A cross-sectional survey was conducted among 218 caregivers of young adolescents (10–14 years) in Mbarara district of south-western Uganda in January and February 2020. Participants were selected through consecutive sampling. A structured, pre-tested questionnaire administered by interviewers was used for data collection. The surveys were computer-assisted using Kobo Collect software. Data was exported to STATA 14 for analysis. Level of SRH communication was measured based on 10 SRH communication topics, while comfort was based on 9 SRH discussion topics. Bivariate and multivariate linear regression analyses were conducted to determine correlates of level of, and comfort with, SRH communication *P*-value < 0.05 was considered for statistical significance.

**Results:**

The mean number of topics that caregivers discussed was 3.9 (SD = 2.7) out of the 10 SRH topics explored. None of the respondents discussed all the topics; 2% reported ever discussing nine topics with their young adolescent, while 3.5% reported never discussing any of the topics. General health and bodily hygiene (89.9%) and HIV/AIDS and other sexually transmitted infections (STIs) (77.5%) were the most commonly discussed, while night emissions in boys (4.3%) and condoms (8.3%) were least discussed. The majority of caregivers (62%) reported a high level of comfort with discussing SRH. The mean comfort score was 21.9 (SD = 3.8). In general, the level of SRH communication increased with an increase in comfort with SRH communication β = 0.22 (0.04); 95% CI = (0.15, 0.30). The level of comfort with SRH communication decreased with an increase in the number of YAs in a household β = -0.92 (0.38); 95%CI = (-1.66,-0.18).

**Conclusion:**

Overall, the level of SRH communication is low and varies according to the number of SRH topics. Caregivers’ comfort with SRH communication with YAs was a significant correlate of SRH communication. This justifies the need for interventions that aim to improve caregivers’ comfort with communicating with young adolescents about SRH.

**Supplementary Information:**

The online version contains supplementary material available at 10.1186/s12889-022-14561-3.

## Plain English summary

Communication between parents and children about sexual and reproductive health (SRH) during the early adolescence stage (10–14 years) plays a significant role in creating positive reproductive health outcomes in later adolescence and adulthood. We conducted surveys in rural areas of Mbarara district among 218 pairs of caregivers and their children aged 10–14 years between January and February 2020. The purpose of the study was to understand whether there is communication between the two, and how comfortable they felt having discussions about SRH. We also asked caregivers and their children about their knowledge of SRH, and how they generally felt about SRH for young people. This study, however, reports only results from caregivers.

We found that, on average, caregivers discussed 4 of the 10 SRH topics explored in this survey with their child, and that the majority of the caregivers reported being very comfortable discussing SRH—especially general health and bodily hygiene—with their children. On the whole, we found that caregivers’ SRH communication was largely influenced by their comfort with discussing SRH with their adolescent child. The level of comfort was influenced by the number of young adolescents living in a household. However, there is no clear justification for this finding from existing literature.

In conclusion, there is a need for interventions that facilitate improvement of communication on SRH between parents/caregivers and young adolescents. These should focus on improving comfort levels by promoting communication skills for caregivers and emphasizing value clarification.

**Supplementary Information:**

The online version contains supplementary material available at 10.1186/s12889-022-14561-3.

## Introduction

The sexual and reproductive health (SRH) of young adolescents (YAs: 10–14 years) is an emerging public health priority in developing countries. YAs comprise about half of the 1.2 billion adolescents aged 10–19 years globally [1]. Young adolescence is often regarded as a relatively healthy phase compared to other age groups [2]. Nevertheless, it is a period of profound changes characterized by the onset of puberty, which comes with physical, emotional, social and cognitive changes that affect their well-being, as well as their sense of self and self-esteem, and the ability to assess risks and consequences [2].

Previous research indicates that puberty accelerates risk-taking among YAs [3, 4]. At this stage of their life, YAs are initiating intimate relationships and acts such as kissing, hugging and fondling [5, 6]. Studies have also shown that they are already engaging in sexual activities, including sexual intercourse [5–7].

YAs in developing countries are disproportionately affected by SRH challenges, including coerced or forced sex, early marriage and gender-based violence [1]. These often culminate in early, unintended and unwanted pregnancies and sexually transmitted infections (STIs), including HIV [8]. YAs also lack information, knowledge, skills and cognitive readiness to make informed decisions related to their SRH, including consensual sex, and condom and contraceptive use [5–7]. Furthermore, gender norms that depict boys as virile and girls as weak and vulnerable often intensify these risks [9].

Caregivers play a significant role in socializing and shaping the attitudes of YAs at an early age that are critical to laying the foundations for positive and safe SRH behaviours. This is through practices such as gender socialization and communication about sexuality in general [10, 11]. Blum’s conceptual framework on early adolescence underscores the significant role of caregivers as part of the micro-environment that influences positive SRH outcomes for YAs [12]. Studies have found a strong association between caregiver SRH communication and reduced sexual risk-taking behaviours among adolescents [13], including delayed sexual initiation and safe SRH practices [14]. Other studies further point to the need to start SRH discussions at an early age, and to provide accurate SRH information [15].

There is considerable research into communication between caregivers and children about SRH in sub-Saharan Africa that emphasizes the existence of such communication despite traditional perspectives [16, 17]. This SRH communication is often punitive [15, 17], limited in breadth to comfortable topics such as abstinence [18], and less about broader SRH topics such as prevention of pregnancy through contraception and the use of condoms [18]. Moreover, many caregivers do not approve of YAs engaging in sexual and romantic relationships, since they are deemed too young and, therefore, not ready to receive SRH information [15]. Caregivers are also not in the position to decipher SRH topics, due to cultural and religious dispositions that inhibit explicit discussions about sex [17, 19]. Low self-efficacy of caregivers, as well as uncertainties about the appropriate timing of sexuality communication, impede sexuality communication [15]. Other structural factors such as caregiver–child connectedness [20] and socio-economic factors may influence communication between caregivers and children. Studies also report a substantial variation in caregiver–child communication by gender, with more pronounced communication between mothers and daughters [15, 21].

Current research specifically into caregiver–child communication on SRH emphasizes older adolescents and barely addresses YAs [1]. Moreover, several studies on caregiver–child communication on SRH present evidence on the level and frequency of SRH communication, but hardly any on the level of comfort with discussing SRH with YAs. There is barely any research assessing correlates of SRH communication and comfort with discussing SRH with YAs in settings where sexuality communication is a cultural taboo. Our research presents data derived from a baseline household survey of caregivers and their YAs (10–14 years) in a community-based participatory research project in rural south-western Uganda. The project aims to improve caregiver communication with YAs through a culturally sensitive intervention targeting caregivers. This paper has two objectives: to describe the current level of, and comfort with, caregiver SRH communication with their children, and to identify their correlates.

## Methods

### Study design and setting

A cross-sectional household survey was conducted in January and February 2020 among caregiver–YA dyads in six villages in Rwebishekye parish, Rwanyamahembe sub-county, Kashari county in rural Mbarara district of south-western Uganda. The study community comprised approximately 1,520 households, of which 29% headed by women, and an estimated population of 6,061 people [22]. The community comprised a relatively homogenous and stable population with one main linguistic group, the Banyankore-Bakiga. The community is served by one public health facility (Bwizibwera Health Centre IV), located about 5 km from the furthest village.

### Study population and sample selection

The study sampling frame comprised all households in the study community with YAs (10–14 years) and their caregivers. A community household profiling exercise was conducted at the start of the study and established an estimate of 300 households comprising YAs. The final sample comprised 218 caregiver–YA dyads (436 study participants overall). The sample size was calculated for the effectiveness study for an intervention to improve SRH communication between caregivers and YAs. It allows a moderate change (effect size 0.2) to be measured in good caregiver–adolescent communication between pre- and post-intervention measurements with a power of 0.8 and alpha of 0.05. This required a total sample size of 277 respondents. Accounting for design effect (× 1.3) and drop-out between waves (× 0.2), the required sample size amounted to 432 participants or 216 dyads.

We used consecutive sampling, based on whether a household contained a YA and whether both caregiver and YA were present simultaneously at the time of the survey. For households comprising more than one YA, we considered the oldest. Caregivers were either biological or non-biological. Within the sample, caregivers included biological parents, step-parents, foster parents or relatives, including older siblings entrusted with the greatest responsibility for the daily care and rearing of the child. Eligibility for caregivers included being 18 years or older, consenting to participate in the study, living in the community for the past six months and living with a YA in their household for whom they were the caregiver for the past six months.

### Data collection

The survey was conducted by trained research assistants who were fluent in English and native speakers of Runyankore-Rukiga. Data were collected using a structured, pre-tested questionnaire administered by an interviewer. The surveys were computer-assisted using Kobo Collect software. The interviews with the caregiver and YA were conducted simultaneously but separately in convenient locations to avoid overhearing and to ensure open and truthful responses. The time for completion of the survey varied from one hour to one hour and 15 min. The survey team was coordinated by two team leaders and community leaders, who assisted in identifying the preselected households. There were also three monitors to check the data for consistency and completeness.

### Community advisory board

Given the participatory and sensitive nature of this research project, the survey questionnaire was reviewed by a multidisciplinary team of researchers and the Community Advisory Board (CAB) in December 2019. The CAB comprised community representatives, including caregivers, young people, teachers, community leaders and influential members of the community, as well as religious leaders from the four majority faiths: Catholic, Anglican, Muslim and Pentecostal. The CAB also included representatives of different government entities, including the Ministry of Health, the Ministry of Gender, Labour and Social Development, and the Ministry of Education and Sports. These stakeholders reviewed the data collection tools and provided feedback on the pertinence and clarity of the survey questions.

### Measures

#### Dependent variables

##### SRH communication

Caregiver–YA SRH communication was explored using 10 SRH-related topics indicated in Table 2. The scale is adapted from the parent-adolescent communication scale (PACS) [23]. However this was adapted based on recommendations from the community advisory board (CAB). Caregivers were asked if they had ever had a discussion on any of the 10 topics. They were presented with the statement ‘Have you ever talked to your child about general health and bodily hygiene?’ The response options were ‘Yes’ and ‘No’. The number of topics discussed was summed, frequencies were run for each response, and the level of communication was stratified by dyad. P-values were based on Fisher’s Exact Test, due to the small number of participants in each dyad.

#### Comfort with SRH discussions

Caregiver comfort with SRH communication was explored using nine SRH topics. Caregivers were asked how comfortable they were discussing any of the SRH topics with their YA children. The topics are indicated in Additional file 1: Appendix A. Caregivers were presented with statements such as ‘How comfortable do you feel discussing general health and bodily hygiene with your YA child?’ The response options for caregivers were ‘very comfortable’, ‘somewhat comfortable’, ‘somewhat uncomfortable’ and ‘very uncomfortable.’ The summated composite score for comfort was calculated with a minimum score of 10 and maximum score of 27. The scores were classified based on Bloom’s criteria [24]. These were organized into 3 groups; scores 22–27 (80–100%) were reported as high comfort; scores 16–21.99 (60–79%) were reported as moderate comfort while scores < 16 (< 60%) were reported as low comfort with SRH discussions. This scale had a Cronbach alpha of 0.73. 

#### Independent variables

##### Background characteristics

Information on socio-demographic variables of caregivers, including age, sex, marital status and religious affiliation, was obtained [25]. The questionnaire included questions on the number of YAs living in the household at the time of the survey, dyad type and the parenting structure of the household (single-parent or two-parent household).

##### Household socio-economic status (SES)

This was measured using variables from the Uganda Bureau of Statistics socio-economic survey [25]. Parameters such as water source (location and the time it takes to reach it), housing characteristics and asset ownership were used to measure SES. They were combined into a proxy indicator—wealth index—using principal component analysis [26]. SES was transformed into an overall variable and recoded as low, medium or high.

##### Connectedness between the caregiver and the YA

Connectedness was measured using three subscales. The parent involvement subscale comprised 10 items, and the positive parenting scale comprised 6 items. Both scales were drawn from the Alabama Parenting Questionnaire, whose target audience is caregivers of children aged 6–18 years [27]. This questionnaire measures five dimensions of parenting that are relevant to the etiology and treatment of children’s externalizing problems [27]. Five Likert-type items were used to assess parental involvement and positive parenting—for example, ‘You have a friendly talk with your child.’ The scores were 5 = always, 4 = often, 3 = sometimes, 2 = almost never and 1 = never. The parental expertise and accessibility scale comprised nine items which assessed both the caregivers’ and the adolescents’ perceptions of the caregivers’ expertise, trustworthiness and accessibility. It is intended for early adolescents (11–14 years) but was adapted for male caregivers in this study. Five Likert-type items were used to assess this scale—for example, ‘My child thinks I give good advice.’ The scores were 1 = strongly agree, 2 = moderately agree, 3 = neither agree nor disagree, 4 = moderately disagree and 5 = strongly disagree [27].

##### Attitudes towards SRH issues of YAs

Attitudes were measured using an eight-item scale on a five-point Likert scale. Caregivers were presented with statements such as ‘You approve of your child having a boyfriend or girlfriend.’ The scores were strongly agree, moderately agree, neither agree nor disagree, moderately disagree and strongly disagree. The scale was scored based on the highest and the lowest scores, with a high score indicating a positive attitude and a low score indicating a negative attitude. The scores were reversed to allow a high score to be indicated as a positive attitude. The summated composite score for attitude was calculated with a minimum score of 8 and maximum score of 24. The scores were classified based on Bloom’s criteria [24]. These were organized into 3 groups; scores 19–24 (80–100%) were reported as positive attitude; scores 14–18.99 (60–79%) were reported as neutral while scores < 14 (< 60%) were reported as negative attitude. The scale had a Cronbach alpha of 0.56.

##### SRH knowledge

This was measured through 27 items to assess knowledge on three main sub-topics: puberty (7 questions), HIV/AIDS (13 questions) and pregnancy prevention (7 questions). A summary score was computed, with the highest score indicating a high level of knowledge and the lowest score indicating a low level of knowledge.. The summated composite score for knowledge was calculated with a minimum score of 49 and maximum score of 81. The scores were classified based on Bloom’s criteria [24]. These were organized into 3 groups; scores 65–81 (80–100%) were reported as high knowledge; scores 49–64.99 (60–79%) were reported as moderate knowledge while scores < 49 (< 60%) were reported as low knowledge.

#### Data analysis

Data analysis was performed using STATA 14 (College Station, Texas, USA). Descriptive statistics were used to describe numbers and percentages for the dependent and independent variables. The prevalence of discussion for each of the 10 SRH topics was presented by dyad type. Fischer’s Exact Tests were used to test for the level of significance of the difference in SRH communication across the dyad type for each of the 10 SRH topics (a 5% level of significance was set). The mean score for the number of topics discussed across the dyads was presented. Bivariate analysis was performed between the dependent variables (level of SRH communication and caregivers’ comfort with SRH discussions) and independent variables. The dependent variables were treated as linear variables (they were normally distributed). We conducted hierarchical linear regression analyses to examine the relationship between the dependent variables (level of SRH communication and caregivers’ comfort with SRH discussions) and the independent variables (demographic characteristics of caregivers, household characteristics, level of comfort, attitudes towards SRH, knowledge of SRH and level of connectedness). Separate linear regression models for number of SRH topics discussed and caregivers’ comfort with SRH discussions were run using a manual backward stepwise selection method. Multi-collinearity was tested using variance inflation factors; none of the variables were affected. Results from the bivariate and multivariate linear regression model for predictors of caregiver and YA communication and comfort with SRH communication are reported in Tables 3, 4, 5 and 6, respectively. Results from the bivariate analysis informed which variables to include in the multivariate linear regression model.

## Results

### Participant characteristics

A total of 218 caregivers were enrolled in the study, of which 76% were women. The mean age of the caregivers was 44.9 years (SD = 12.61). Seventy-three per cent were biological caregivers, and the majority (68.3%) had attained primary education, while 11.9% had not received any formal education. The caregiver–YA dyads comprised 96 with female caregiver and daughter (44.0%), 68 male caregiver and daughter (31.2%), 30 female caregiver and son (13.8%) and 24 male caregiver and son 24 (11%). The majority (76.6%) of the households were two-caregiver households, with an average of two YAs (Table 1).

**Table 1:** Socio-demographic characteristics of caregivers (*N* = 218)

Characteristics of caregivers	Categories	n (%)
**Age**	**Mean 44.87 (SD = 12.61)**	-
	< 35 years	44 (20.4)
	35–49 years	103 (47.7)
	50 years +	69 (31.4)
**Sex**	Male	53 (24.3)
	Female	165 (75.7)
**Relationship with YA**	Biological caregiver	160 (73.4)
	Non-biological caregiver	58 (26.6)
**Formal education level**	No formal education	26 (11.9)
	Primary education	149 (68.3)
	Secondary education	32 (14.7)
	Tertiary education	11 (5.1)
**Marital status**	Single	38 (17.4)
	Married	180 (82.6)
**Religion**	Catholic	28 (12.8)
	Anglican	155 (71.1)
	Pentecostal	32 (14.7)
	Muslim	3 (1.4)
**Attendance of religious services**	Never	9 (4.1)
	Once in the past month	15 (6.9)
	Two or three times a month	128 (58.7)
	Once a week or more	66 (30.3)
**Dyad type**	Female caregiver–daughter	96 (44.0)
	Female caregiver–son	68 (31.2)
	Male caregiver–daughter	30 (13.8)
	Male caregiver–son	24 (11.0)
**Parenting structure**	Single-caregiver household	51 (23.4)
	Two-caregiver household	167 (76.6)
**Socio-economic status**	Low	88 (40.4)
	Medium	88 (40.4)
	High	42 (19.3)
**Number of YAs in the household**	Median (IQR)	2 (1,2)

Two participants missing age (age unknown)

### Descriptive analysis

#### Level of SRH communication

Ten SRH topics were explored in the study. None of the respondents discussed all 10 topics. Two per cent of the caregivers reported ever discussing nine SRH topics with the YAs, while 3.5% reported never discussing any topics. The mean number of topics ever discussed was 3.9. Twenty-two per cent of the caregivers reported discussing at least three of the topics, and 7% reported discussing at least one of the topics.

Overall, general health and bodily hygiene was discussed by majority of the dyads (89.9%), followed by HIV/AIDS and other STIs (77.5%). In contrast, only 4.3% of the dyads discussed night emissions in boys. There was no significant difference in communication of SRH topics across the dyads, except for HIV/AIDS and other STIs, which were more likely to be discussed in dyads with female caregivers (*p* < 0.05) (Table 2).

**Table 2:** Descriptive statistics of level of SRH communication by SRH topic across the dyads

SRH topic	Caregiver-reported communication N (%) *N* = 218	Dyad type				
		**Female caregiver–daughter n (%)**	**Female caregiver–son n (%)**	**Male caregiver–daughter n (%)**	**Male caregiver–son n (%)**	***P*-value**
**General health and bodily hygiene**	196 (89.9%)	86 (43.9%)	62 (31.6%)	28 (14.3%)	20 (10.2%)	0.645
**Menstruation and menstrual hygiene**	62 (29.8%)	30 (48.4%)	14 (22.6%)	9 (14.5%)	9 (14.5%)	0.340
**Night emissions in boys**	9 (4.30%)	5 (55.6%)	1 (11.1%)	2 (22.2%)	1 (11.1%)	0.570
**Romantic relationships**	73 (33.5%)	32 (43.8%)	21 (28.8%)	11 (15.1%)	9 (12.3%)	0.915
**Handling sexual pressure**	130 (59.6%)	57 (43.9%)	42 (32.3%)	16 (12.3%)	15 (11.5%)	0.871
**HIV and other STIs**	169 (77.5%)	85 (50.3%)	49 (29.0%)	19 (11.2%)	16 (9.5%)	**0.003**
**Birth control**	34 (15.6%)	16 (47.0%)	9 (26.5%)	4 (11.8%)	5 (14.7%)	0.804
**Condoms**	18 (8.26%)	9 (50.0%)	4 (22.2%)	1 (5.6%)	4 (22.2%)	0.276
**Sexual conduct**	43 (19.7%)	22 (51.2%)	10 (23.3%)	6 (13.9%)	5 (11.6%)	0.633
**Sexual violence and reporting**	91 (41.7%)	39 (42.9%)	30 (32.9%)	14 (15.4%)	8 (8.8%)	0.752
**Mean number of topics discussed**	**3.9 (2.1)**	**4.1 (2.1)**	**3.7 (1.9)**	**3.9 (2.2)**	**3.9 (2.7)**	**0.85**

Significant difference at *p* < 0.05 across four dyads

#### Caregivers’ comfort with SRH discussions with young adolescents

The majority of the caregivers (63.4%) reported a high level of comfort with SRH discussions with YAs, 31.7% were moderately comfortable, and 4.9% reported a low level of comfort. There was a higher level of comfort among female caregivers (63%) than among male caregivers, but the difference was not statistically significant (*p* > 0.05). General health and bodily hygiene was the most comfortable topic, followed by HIV/AIDS and other STIs. Having babies, birth control and night emissions in boys were the least comfortable topics (Additional file 1: Appendix A).

#### Attitudes towards SRH issues of young adolescents

The majority of the caregivers (84.8%) had a negative attitude towards SRH issues of YAs, while only about 0.7% of the caregivers had a positive attitude, and 14.5% had a moderate attitude. Female caregivers (85%) reported a significantly higher negative attitude towards SRH issues of YAs compared to male caregivers (*p* = 0.05). The median score for attitude was 11 out of a maximum of 12.

#### SRH knowledge of caregivers

Fifteen per cent of the caregivers reported a high level of knowledge of SRH, while 3 per cent reported a low level of knowledge. The vast majority (84%) of the caregivers reported a moderate level of SRH knowledge. Female caregivers had greater knowledge than male caregivers, though this was not statistically significant.

#### Connectedness between caregivers and young adolescents

Sixteen per cent of the caregivers reported a high level of connectedness with YAs, while 34% reported a low level of connectedness. Around half (49%) reported a medium level of connectedness. Female caregivers reported a higher level of connectedness than male caregivers. Connectedness was measured through three subscales: caregiver involvement, positive parenting, and parental expertise and accessibility. A third (33%) of the caregivers reported a high level of involvement, with the majority of these being female. Over a third (39%) of the caregivers reported a high level of positive parenting, while 32% reported a high level of parental expertise and accessibility.

#### Correlates of level of SRH communication between caregivers and young adolescents

Bivariate linear regression was carried out to investigate the relationship between socio-demographic characteristics of the caregivers, comfort with SRH communication, attitudes towards SRH, level of connectedness and knowledge of SRH with SRH communication (Table 3). The analysis indicated a significant (*p* < 0.001) positive linear relationship between comfort with SRH discussions and level of SRH communication. A unit of increase in comfort with SRH discussions increases SRH communication by 0.25 units (SE = 0.04). On the other hand, the level of SRH communication reduced with an increase in the number of YAs in a household (-0.45, SE = 0.19; *p* < 0.05).

**Table 3:** Correlates of caregiver and young adolescent SRH communication

Variable	Unadjusted β and SE	95% confidence interval	*P*-value
Sex			
Female	1.0		
Male	-0.01 (0.34)	(-0.69, 0.66)	0.967
Age	1.0		
Mean 44.87 (12.6)	-0.01 (0.21)	(-0.44, 0.41)	0.953
Educational attainment			
No formal education	1.0		
Primary education	0.46(0.46)	(-0.44,1.36)	
Secondary education	0.55 (0.57)	(-0.57, 1.68)	0.751
Tertiary education	0.27(0.85)	(-1.41, 1.95)	
Marital status			
Single	1.0		
Married	-0.17 (0.39)	(-0.95, -0.61)	0.669
Relationship with YA			
Biological caregiver	1.0		
Non-biological caregiver	0.48 (0.32)	(-0.07, 1.18)	0.149
Parenting structure			
Single-caregiver household	1.0		
Two-caregiver household	-0.57 (0.35)	(-1.27, 0.12)	0.106
Religion			
Catholic	1.0		
Anglican	-0.13 (0.45)	(-1.01, 0.76)	
Pentecostal	-0.53 (0.57)	(-1.64, 0.59)	0.553
Muslim	-1.41 (1.28)	(-3.93, 1.11)	
Number of religious services			
Never	1.0		
Once a week	-0.4 (0.79)	(-1.95, 1.16)	0.579
2–3 times a month	-0.72 (0.77)	(-2.23, 0.79)	
Once in the past month	-0.96 (0.94)	(-2.82, 0.89)	
Dyad type			
Female caregiver–daughter	1.0		
Female caregiver–son	-0.41 (0.35)	(-1.10, 0.27)	
Male caregiver–daughter	-0.20 (0.46)	(-1.10, 0.70)	0.700
Male caregiver–son	-0.10 (0.50)	(-1.10, 0.89)	
Number of YAs per household			
Mean/SD	-0.45 (0.19)	(**-**0.82, -0.78)	**0.018**
Socio-economic status			
High	1.0		
Moderate	-0.23 (0.41)	(-1.03, 0.58)	0.245
Low	0.32 (0.41)	(-0.48, 1.13)	
Caregiver–child connectedness	0.17 (0.12)	(-0.08, 0.41)	0.177
Caregiver involvement	-0.07 (0.04)	(-0.14, 0.00)	0.068
Positive parenting	-0.04 (0.06)	(-0.17, 0.08)	0.482
Parental expertise and accessibility	0.00 (0.06)	(-0.12, 0.12)	0.959
Attitudes towards SRH of YAs	-0.01 (0.07)	(-0.15, 0.13)	0.932
SRH knowledge	-0.00 (0.04)	(-0.07, 0.07)	0.997
Caregiver comfort with SRH discussions	0.25 (0.04)	(0.18, 0.32)	**0.000**

In the multivariate linear regression analysis, we ran three models using the manual backward stepwise approach to identify the variables significantly predicting SRH communication. We considered variables that were statistically significant in the bivariate analysis (level of comfort with SRH communication and number of YAs in a household), those with a borderline p-value (caregiving structure) and those that indicate biological plausibility (based on previous findings on predictors of caregiver–child communication on SRH) (Table 4). These included caregiver involvement, sex and relationship type. The overall regression was statistically significant (*R*^2^ = 0.23, F (7,183) = 7.61; *p* < 0.001. It was found that the level of comfort with SRH communication significantly predicted the level of SRH communication (0.22 (0.04); *p* > 0.001).

**Table 4:** Multivariate correlates of SRH communication

Variable	Adjusted β (SE)	95% confidence interval	*R*^2^	Number of observations	*P*-value
Model 1			***R***^**2**^** = 0.22** ***P* < 0.000**	194	
Number of YAs in a household	-0.33 (0.17)	(-0.67, 0.12)			0.059
Caregiver comfort with SRH discussions	0.24 (0.36)	(0.17, 0.31)^**^		-	0.000
Model 2			***R***^**2**^** = 0.22** ***P* < 0.000**	194	
Comfort level of SRH discussions	0.24 (0.04)	(0.17, 0.31)^**^			0.000
Number of YAs in a household	-0.31 (0.17)	(-0.65, 0.03)			0.073
Model 3			***R***^**2**^** = 0.23** ***P* < 0.000**	191	
Comfort level of SRH discussions	0.22 (0.04)	(0.15,0.3)			0.000
Number of YAs in a household	-0.34 (0.18)	(-0.70, 0.02)			0.07
Sex					
Female	1.0				
Male	-0.38 (0.33)	(-1.02, 0.26)			0.246
Caregiver involvement					
High	1.0				
Moderate	0.40 (0.32)	(-0.23, 1.03)			0.213
Low	0.50 (0.38)	(-0.24, 1.24)			1.188
Relationship type					
Biological caregiver	1.0				
Non-biological caregiver	0.12 (0.33)	(-0.53, 0.76)			0.73

#### Correlates of level of comfort with SRH communication between caregivers and young adolescents

The bivariate analysis for level of comfort with SRH communication indicated that the number of YAs in a household (-0.98, SE = 0.34) significantly predicted comfort with SRH communication, although it had a negative correlation (Table 5). The more the YAs in a given households, the less comfortable a caregiver felt discussing SRH.

**Table 5:** Correlates of caregivers’ comfort with SRH communication with young adolescents

Characteristics	Unadjusted β (SE)	95% confidence interval	*P*-value
Sex			
Female	1.0		
Male	0.49 (0.61)	(-0.71, 1.68)	0.424
Age	0.07 (0.37)	(-0.66, 0.79)	0.847
Educational attainment			
No formal education	1.0		
Primary education	-0.33 (0.84)	(-1.99, 1.33)	
Secondary education	-0.32 (1.03)	(-2.36, 1.71)	
Tertiary education	0.65 (1.39)	(-2.09, 3.39)	0.852
Marital status			
Single	1.0		
Married	0.14 (0.69)	(-1.22, 1.49)	0.842
Relationship with YA			
Biological caregiver	1.0		
Non-biological caregiver	0.68 (0.59)	(-0.48, 1.85)	0.249
Parenting structure			
Single-caregiver household	1.0		
Two-caregiver household	-0.80 (0.61)	(-2.01, 0.41)	0.192
Religion			
Catholic	1.0		
Anglican	-0.16 (0.80)	(-1.74, 1.42)	
Pentecostal	-1.95 (1.00)	(-3.93, 0.03)	
Muslim	1.02 (2.30)	(-3.51, 5.56)	0.089
Number of religious services			
Never	1.0		
Once a week	0.53 (1.36)	(-2.16, 3.21)	
Two or three times a month	0.89 (1.32)	(-1.71, 3.49)	0.827
Once in the past month	0.31 (1.61)	(-2.87, 3.49)	
Dyad type			
Female caregiver–daughter	1.0		
Female caregiver–son	0.01 (0.61)	(-1.20, 1.21)	
Male caregiver–daughter	0.08 (0.81)	(-1.52, 1.68)	0.693
Male caregiver–son	-0.98 (0.87)	(-2.70, 0.74)	
Number of YAs in a household	-0.98 (0.34)	(-1.65, -0.31)	0.0041
Caregiver–child connectedness			
High	1.0		
Moderate	0.44 (0.82)	(-1.17, 2.04)	0.194
Low	-0.72 (0.86)	(-2.41, 0.97)	
Caregiver involvement			
High	1.01.0		
Moderate	0.89 (0.59)	(-0.29, 2.06)	0.327
Low	0.42 (0.71)	(-0.98, 1.81)	
Positive parenting			
High	1.0		
Moderate	0.32 (0.56)	(-0.79, 1.44)	
Low	-0.53 (0.82)	(-2.15, 1.09)	0.549
Parental expertise and accessibility			
High	1.0		
Moderate	-0.07 (0.62)	(-1.27, 1.15)	0.969
Low	0.47 (2.30)	(-4.08, 5.01)	
Attitudes towards SRH of YAs			
High	1.0		
Moderate	-4.50 (3.66)	(-11.73, 2.72)	
Low	-3.66 (3.62)	(-10.80, 3.48)	0.283
SRH knowledge			
High	1.0		0.668
Moderate	-1.66 (2.21)	(-6.01, 2.69)	
Low	-2.00(2.29)	(-6.52, 2.52)	

We ran three models using the manual backward stepwise approach. In the final model, we considered the variables that were statistically significant in the bivariate analysis and also included those that were biologically plausible, as well as borderline p-value (religion and sex) (Table 6). The overall regression was not statistically significant (*R*^2^ = 0.09, F (9,169) = 1.84; *p* = 0.06). However, it was found that the number of YAs in a household significantly predicted the level of SRH communication (*p* < 0.05).

**Table 6:** Multivariate analysis for correlates of caregivers’ comfort with SRH communication with young adolescents

Variable	Adjusted β (SE)	95% confidence interval	*R*^2^	Number of observations	*P*-value
Model 1			***R***^**2**^** = 0.07** ***P* < 0.008**	213	
Number of YAs in a household	-0.95 (0.34)	(-1.62, -2.29)			0.005
Religion					
Catholic					
Anglican	0.00 (0.79)	(-1.56, 1.56)			1.000
Pentecostal	-1.79 (0.99)	(-3.75, 0.17)			0.073
Muslim	1.65 (2.27)	(-2.82, 6.13)			0.468
Sex					
Female	0.63 (0.60)	(-0.54, 1.81)			0.291
Male					
Model 2			***R***^**2**^** = 0.09** ***P* = 0.01**	182	
Number of YAs in a household	-0.92 (0.37)	(-1.66, -0.19)			0.014
Religion					
Catholic					
Anglican	-0.38 (0.86)	(-2.09,1.32)			0.655
Pentecostal	-2.55 (1.10)	(-4.72, -0.38)			0.022
Muslim	1.26 (2.34)	(-3.35, 5.86)			0.591
Sex					
Female					
Male	0.23 (0.65)	(-1.06, 1.53)			0.720
Caregiver–child connectedness					
High					
Moderate	0.25 (0.81)	(-1.35, 1.85)			0.759
Low	-0.92 (0.84)	(-2.58, 0.73)			0.273
Model 3			*R*^2^ = 0.09 *P* = 0.06		
Number of YAs in a household	-0.92 (0.38)	(-1.66, -0.18)			0.016
SRH knowledge					
High					
Moderate	-3.98 (3.87)	(-11.62, 3.65)			0.304
Low	-4.25 (3.94)	(-12.03, 3.52)			0.282
Sex					
Female					
Male	0.19 (0.67)	(-1.12, 1.51)			0.774
Connectedness					
High					
Moderate	-0.01 (0.82)	(-1.64, 1.62)			0.991
Low	-1.05 (0.85)	(-2.73, 0.63)			0.218
Religion					
Catholic					
Anglican	-0.29 (0.87)	(-2.01, 1.44)			0.744
Pentecostal	-2.40 (1.13)	(-4.63, -0.19)			0.034
Muslim	1.32 (2.34)	(-3.29, 5.94)			0.573

## Discussion

This study sought to assess the current level of communication between caregivers and YAs about SRH, and caregivers’ comfort with such discussions, and identify their correlates. The study was conducted in a rural community in south-western Uganda, where an intervention to improve communication between caregivers and YAs on SRH would be tested. Unlike many studies which focus on SRH communication with older adolescents, this study focuses on YAs aged 10–14 years. This approach is driven by the notion that young adolescence is a stage of transition from childhood to adulthood where critical changes occur, especially in terms of sexual development [2]. Addressing SRH issues during this transitional phase is considered to have more positive outcomes than dealing with them later in life. However, there is also building evidence the risk-taking behaviours is already occurring at this age [7].

Overall, our findings indicate that communication about SRH does take place between caregivers and their YAs. However, this was relatively rare and varied according to the topics discussed. On average, 21.6% of caregivers in the study reported ever discussing an average of 3.9 of the 10 SRH topics listed in the questionnaire. This finding falls in tandem with several other studies in similar settings—for example, a study conducted in Korogocho settlement in western Kenya indicated that communication between caregivers and very young adolescents was rare [15]. Similar findings are reported in a study conducted in Zanzibar, where only 40% of caregivers had ever communicated with their children about SRH [18]. However, the latter study reports communication about SRH with older adolescents (aged 15–19).

A considerable number of caregivers reported discussing general health and bodily hygiene, and HIV/AIDS and other STIs. Indeed, on the comfort scale, caregivers reported high levels of comfort discussing HIV/AIDS and other STIs, as well as general health and bodily hygiene. Notwithstanding is the major finding of this study that the number of SRH topics discussed increased with an increasing level of comfort with SRH discussions. The probable reason for high reports of discussions on general health and bodily hygiene is that these topics can be discussed with minimal embarrassment. As far as HIV/AIDS is concerned, excess messaging around HIV/AIDS in the media, coupled with the high risk perception of HIV infection in many communities, may have triggered a lot of discussion around this topic. Topics deemed to be sensitive—such as night emissions in boys, condoms, birth control and sexual conduct—were discussed the least. Low or moderate levels of knowledge and a high proportion of caregivers reporting a negative attitude towards SRH in our findings could account for the low level of discussion of these latter topics. Additionally, evidence also shows that parents associate discussions with adolescents about condoms and birth control with being comparable to encouraging them to engage in sexual intercourse [28].

The relationship between the SRH topics most and least commonly discussed and their perceived sensitivity strongly justifies the finding that the level of SRH communication increases with increasing level of comfort. This interrelates with the notion that open discussions about sexual issues are a taboo in many African settings, and the fact that many caregivers believed that it was too early to begin initiating discussions about sex [15]. These factors, though not addressed in this study, serve as proxies for SRH communication by influencing how comfortable caregivers feel discussing these SRH topics with YAS. Our findings specifically reveal that religion and the number of adolescents in a household influence caregivers’ comfort with SRH communication. In their review, Abdallah et al. (2017) report religion as one of the factors influencing SRH communication in East Africa [29].

Although there was no significant difference in SRH communication across the dyad types for each of the SRH topics except for HIV/AIDS and other STIs, mother–daughter dyads were reported to have the highest mean number of topics discussed, while mother–son dyads were reported to have the lowest mean number of topics. Many studies implicate the influence of gender on caregiver–child communication, with mothers communicating more than fathers, and girls receiving more communication than boys. Girls are disproportionately vulnerable to SRH risks than boys, and mothers spend more time with children than fathers [29]. Moreover, evidence indicates that mothers are the preferred partners for socializing their daughters about sexuality [19, 29, 30].

We found that the level of comfort with SRH communication reduced with an increase in the number of YAs in a household. It is possible that caregivers may find it uncomfortable having SRH discussions with more children than it would be if they were fewer. Previous studies have particularly investigated the effect of family size on the level of SRH communication. In this study, the number of YAs in a given household could serve as an indicator for family size. Studies in Bangladesh and Ethiopia indicate that the bigger the family size, the lower the level of SRH communication [31, 32]. Another study in Ethiopia reports no association between family size and the level of SRH communication [33]. Zakaria et al. attributes their findings to presence of older siblings in the household that the adolescents would most likely prefer to talk to about their SRH issues rather than their parents [31]. Muhwezi et al. reveals that adolescents preferred to talk to their siblings about SRH than their parents because their parents were not comfortable about these SRH discussions [34]. The other reason given for not discussing SRH issues in larger families could be due to parents feeling overburdened by the number of children to speak to and that parents are less concerned for SRH communication as the family size increases [32]. However, there is need for further research to explore the association between comfort with SRH communication and family size.

## Conclusion

The results of this study suggest that SRH communication between caregivers and YAs was low. SRH communication was also found to increase with increase in comfort with SRH communication. We also found that the more the YAs in a household, the lower the level of SRH communication. Comfort of SRH communication was found to reduce with an increase in number of YAs in a household. These findings provide a basis for interventions to improve communication between caregivers and children. First, training on value clarification and communication skills that enable caregivers to discuss SRH topics with less embarrassment and create a predisposition towards a positive attitude towards YA SRH is important. Topics focusing on general parenting skills—particularly the quality of their relationships—with the assumption that it inculcates positive caregiver–child relationships, would facilitate and increase the level of comfort with SRH discussions. There is a need for qualitative studies to gain a deeper understanding of determinants of comfort with discussing SRH with YAs. 

### Study limitations

Limitations of our study include a relatively small sample size, which affects the power of the study. This affects comparison of SRH communication by gender, yet evidence highlights its important influence on SRH communication [15, 21]. The samples for males are generally too small to make a substantial comparison.

## Supplementary Information

**Additional file 1:** **Appendix A.** Level of comfort of SRH communication. **Appendix B.** SRH communication by number of topics. **Appendix C.** Caregiver attitudes towards SRH of Adolescents. **Appendix D.** Comfort of SRH Discussions by dyad type. **Appendix E.** Distribution of independent variables by sex of the caregiver.

## Data Availability

All the data needed for this manuscript have been included. In case there is a need for clarification, the corresponding author can be contacted. Datasets used to analyse the data are available on request.

## Abbreviations

AIDS: Acquired Immunodeficiency Syndrome
CAB: Community Advisory Board
HIV: Human Immunodeficiency Virus
SRH: Sexual and reproductive health
STI: Sexually transmitted infection
YA: Young adolescent
