# Supplementary material for: Level and comfort of caregiver–young adolescent communication on sexual and reproductive health: a cross-sectional survey in south-western Uganda
Source: BMC Public Health. 2022 Nov 19;22:2129. doi: 10.1186/s12889-022-14561-3 (PMC9675188; doi:10.1186/s12889-022-14561-3)
Supplement: Supplementary file 1 — Additional file 1: Appendix A. Level of comfort of SRH communication. Appendix B. SRH communication by number of topics. Appendix C. Caregiver attitudes towards SRH of Adolescents. Appendix D. Comfort of SRH Discussions by dyad type. Appendix E. Distribution of independent variables by sex of the caregiver. [file 12889_2022_14561_MOESM1_ESM.docx]

**Additional file 1**

**Appendix A:** Level of comfort of SRH communication

| SRH Topic | Scale | | | |
| --- | --- | --- | --- | --- |
|  | Very Comfortable | Somewhat comfortable | Somewhat uncomfortable | Very uncomfortable |
| General Health and body hygiene | 197 (90.4%) | 13 (6.0%) | 3 (1.4%) | 5 (2.3%) |
| Menstruation and menstruation hygiene | 144 (66.1%) | 13 (6.0%) | 7 (3.2%) | 31 (14.2%) |
| For girls; pubic hair, soft voice, breasts | 134 (61.5%) | 23 (10.6%) | 6 (2.8%) | 36 (16.5%) |
| For boys; wet dreams, pubic hair, enlarged private parts, deep voice | 101 (46.3%) | 28 (12.8%) | 12 (5.5%) | 48 (22.0%) |
| HIV/AIDS, transmission and prevention and other STIs | 171 (78.4%) | 24 (11.0%) | 3 (1.4%) | 20 (9.2%) |
| Sexual relationships | 132 (60.6%) | 34 (15.6%) | 8 (3.7%) | 44 (20.2%) |
| Romantic relationships | 116 (53.2%) | 30 (13.8%) | 15 (6.9%) | 55 (25.2%) |
| Having babies and Birth control | 71 (32.6%) | 29 (13.3%) | 12 (5.5%) | 99(45.4%) |
| Sexual pressure by peers and potential Sexual partners | 156(71.6%) | 29 (13.3%) | 1(0.5%) | 31 (14.2%) |

**Appendix B:** SRH communication by number of topics

| **No. of topics** | **No. of parents ever discussed** | **Percentage** |
| --- | --- | --- |
| 0 | 7 | 3.5 |
| 1 | 14 | 7.0 |
| 2 | 33 | 16.6 |
| 3 | 43 | 21.6 |
| 4 | 28 | 14.1 |
| 5 | 35 | 17.6 |
| 6 | 13 | 6.5 |
| 7 | 12 | 6.0 |
| 8 | 10 | 5.0 |
| 9 | 4 | 2.0 |

**Appendix C:** Caregiver attitudes towards SRH of Adolescents.

|  | **Response Key** | **Disagree (%)** | **Not certain (%)** | **Agree (%)** |
| --- | --- | --- | --- | --- |
| 1.1 | You would approve of your child having a boyfriend or girlfriend. | 93.6 | 0.05 | 5.9 |
| 1.2 | It is ok for young adolescent *girls* (10-14) years to engage in sexual relationships. | 99.5 | 0 | 0.5 |
| 1.3 | It is ok for young adolescent *boys* (10-14) years to engage in sexual relationships. | 97.9 | 0.5 | 1.5 |
| 1.4 | It is ok for young adolescent *girls* to have access to condoms. | 94.7 | 1.5 | 3.9 |
| 1.5 | It is ok for young adolescent *boys* to have access to condoms. | 92.8 | 2.1 | 5.2 |
| 1.6 | A young adolescent girl who is sexually active should be allowed to use contraceptives. | 84.7 | 0.5 | 14.8 |
| 1.7 | A *girl* who becomes pregnant should be allowed to continue school. | 45.4 | 1.0 | 53.7 |
| 1.8 | A school *boy* who impregnates a girl should be allowed to continue school. | 20.3 | 5.6 | 74.1 |

**Appendix D:** Comfort of SRH Discussions by dyad type

| Topic | Total | Female Caregiver-Daughter  n (%) | Female caregiver-son  n (%) | Male Caregiver-Daughter  n (%) | Male caregiver-son  n (%) | P-Value |
| --- | --- | --- | --- | --- | --- | --- |
| General Health and body hygiene |  |  |  |  |  |  |
| Very Comfortable | 197 (90.4) | 86 (89.6) | 62 (91.2) | 28 (93.3) | 21 (87.5) | **0.55** |
| Somewhat comfortable | 13 (6.0) | 8 (8.33) | 3 (4.4) | 1 (3.3) | 1 (4.2) |  |
| Somewhat uncomfortable | 3 (1.4) | 1 (1.04) | 1 (1.5) | 1 (3.3) | 0 (0.0) |  |
| Very uncomfortable | 5 (2.3) | 1 (1.04) | 2 (2.9) | 0 (0.0) | 2 (8.3) |  |
| Menstruation and menstruation hygiene |  |  |  |  |  |  |
| Very Comfortable | 144 (66.1%) | 63 (65.6) | 41 (60.3) | 25 (83.3) | 15 (62.5) | **0.89** |
| Somewhat comfortable | 13 (6.0%) | 6 (6.3) | 5 (7.4) | 1 (3.3) | 1 (4.2) |  |
| Somewhat uncomfortable | 7 (3.2%) | 3 (3.1) | 3 (4.4) | 1 (3.3) | 0 (0.0) |  |
| Very uncomfortable | 31 (14.2%) | 13 (13.5) | 12 (17.7) | 1 (3.3) | 5 (20.8) |  |
| *For girls;* pubic hair, soft voice, breasts |  |  |  |  |  |  |
| Very Comfortable | 134 (61.5%) | 61 (63.5) | 39 (57.4) | 21(70.0) | 13 (54.2) | **0.95** |
| Somewhat comfortable | 23 (10.6%) | 11 (11.5) | 8 (11.8) | 2 (6.7) | 2 (8.3) |  |
| Somewhat uncomfortable | 6 (2.8%) | 4 (4.2) | 1 (1.5) | 1 (3.3) | 0 (0.0) |  |
| Very uncomfortable | 36 (16.5%) | 12 (12.5) | 14 (20.6) | 4 (13.3) | 6 (25.0) |  |
| *For boys;* wet dreams, pubic hair, enlarged private parts, deep voice |  |  |  |  |  |  |
| Very Comfortable | 101 (46.3%) | 43 (44.8) | 32 (47.1) | 15 (50.0) | 10 (41.7) | **0.91** |
| Somewhat comfortable | 28 (12.8%) | 13 (13.5) | 8 (11.8) | 4 (13.3) | 3 (12.5) |  |
| Somewhat uncomfortable | 12 (5.5%) | 8 (8.3) | 3 (4.4) | 1 (3.3) | 0 (0.0) |  |
| Very uncomfortable | 48 (22.0%) | 22 (22.9) | 15 (22.1) | 6 (25.0) | 6 (25.0) |  |
| HIV/AIDS, transmission and prevention and other STIs |  |  |  |  |  |  |
| Very Comfortable | 171 (78.4%) | 74 (77.1) | 57 (83.8) | 21 (70.0) | 19 (79.2) | **0.39** |
| Somewhat comfortable | 24 (11.0%) | 13 (13.5) | 7 (10.3) | 3 (10.0) | 1 (4.2) |  |
| Somewhat uncomfortable | 3 (1.4%) | 2 (2.1) | 0 (0.0) | 1 (3.3) | 0 (0.0) |  |
| Very uncomfortable | 20 (9.2%) | 7 (7.3) | 4 (5.9) | 5 (16.7) | 4 (16.7) |  |
| Sexual relationships |  |  |  |  |  |  |
| Very Comfortable | 132 (60.6%) | 58 (60.4) | 45 (66.2) | 16 (53.3) | 13 (54.2) | **0.61** |
| Somewhat comfortable | 34 (15.6%) | 16 (16.7) | 10 (14.7) | 5 (16.7) | 3 (12.5) |  |
| Somewhat uncomfortable | 8 (3.7%) | 6 (6.3) | 1 (1.5) | 1 (3.3) | 0 (0.0) |  |
| Very uncomfortable | 44 (20.2%) | 16 (16.7) | 12 (17.7) | 8 (26.7) | 8 (33.3) |  |
| Romantic relationships |  |  |  |  |  |  |
| Very Comfortable | 116 (53.2%) | 47 (48.9) | 43 (63.2) | 17 (56.7) | 9 (37.5) | **0.54** |
| Somewhat comfortable | 30 (13.8%) | 14 (14.6) | 7 (10.3) | 4 (13.3) | 5 (20.8) |  |
| Somewhat uncomfortable | 15 (6.9%) | 10 (10.4) | 2 (2.94) | 2 (6.7) | 1 (4.2) |  |
| Very uncomfortable | 55 (25.2%) | 24 (25.0) | 15 (22.1) | 7 (23.3) | 9 (37.5) |  |
| Having babies and Birth control |  |  |  |  |  |  |
| Very Comfortable | 71 (32.6%) | 34 (35.4) | 23 (33.8) | 6 (20.0) | 8 (33.3) | **0.22** |
| Somewhat comfortable | 29 (13.3%) | 17 (17.7) | 5 (7.35) | 5 (16.7) | 1 (4.2) |  |
| Somewhat uncomfortable | 12 (5.5%) | 3 (3.13) | 4 (5.88) | 2 (6.7) | 4 (16.7) |  |
| Very uncomfortable | 99(45.4%) | 39 (40.6) | 33 (48.5) | 17 (15.7) | 10 (41.7) |  |
| Sexual pressure by peers and potential Sexual partners |  |  |  |  |  |  |
| Very Comfortable | 156(71.6%) | 66 (68.8) | 57 (83.8) | 18 (60.0) | 15 (62.5) | **0.07** |
| Somewhat comfortable | 29 (13.3%) | 14 (14.6) | 6 (8.8) | 7 (23.3) | 2 (8.33) |  |
| Somewhat uncomfortable | 1(0.5%) | 1 (1.04) | 0 (0.0) | 0 (0.0) | 0 (0.0) |  |
| Very uncomfortable | 31 (14.2%) | 15 (15.6) | 5 (7.4) | 5 (16.7) | 6 (25.0) |  |

**APPENDIX E:** **Distribution of independent variables by sex of the caregiver**

| **Parameter** | **Female Caregiver-n(%)** | **Male caregiver-n (%)** | **N (%)** | **P-value** |
| --- | --- | --- | --- | --- |
| **Connectedness**  High  Moderate  Low | 21(15)  76 (54.3)  43 (30.7) | 9 (19.2)  17 (36.2)  21 (44.7) | 30 (16.0)  93 (49.7)  64 (34.2) | 0.09 |
| **Caregiver involvement**  High  Moderate  Low | 52 (31.9)  77 (47.2)  34 (20.9) | 19 (36.5)  19 (36.5)  14 (26.9) | 71 (33.0)  96 (44.7)  48 (22.3) | 0.38 |
| **Positive parenting**  High  Moderate  Low | 65 (39.4)  79 (47.9)  21 (12.7) | 20 (37.7)  25 (47.2)  8 (15.1) | 85 (38.9)  104 (47.7)  29 (13.3) | 0.90 |
| **Parental expertise and accessibility**  High  Moderate  Low | 41 (28.9)  99 (69.7)  2 (1.4) | 21 (44.7)  25 (53.2)  1 (2.13) | 62 (32.8)  124 (65.6)  3 (1.6) | 0.12 |
| **Attitude towards YA SRH**  Positive  Neutral  Negative | 1 (0.72)  20 (14.5)  117 (84.8) | 0 (0.0)  13 (30.9)  29 (69.1) | 1 (0.6)  33 (18.3)  146 (81.1) | 0.05 |
| **Knowledge of SRH**  High  Moderate  Low | 2 (1.23)  137 (84.1)  24 (14.7) | 1 (2.0)  43 (84.3)  7 (13.7) | 31 (14.5)  180 (84.1)  3 (1.4) | 0.92 |
| **Comfort of SRH discussion**  High  Moderate  Low | 102 (63.4)  51 (31.7)  8 (4.9) | 30 (57.7)  15 (28.9)  7 (13.5) | 132 (62.0)  66 (31.0)  15 (7.0) | 0.11 |

;
